# Specialist care visits outside the hospital by South Australian older adults

**DOI:** 10.1186/s12913-024-11268-6

**Published:** 2024-07-11

**Authors:** Dennis Asante, Williams Agyemang-Duah, Paul Worley, Gloria Essilfie, Vivian Isaac

**Affiliations:** 1 College of Medicine & Public Health, Rural and Remote Health, Flinders University, Renmark, South Australia; 2 Department of Geography and Planning, Queen’s University, Kingston, ON K7L 3N6 Canada; 3 Riverland Mallee Coorong Local Health Network, SA Health, Government of South Australia, Berri, South Australia; 4 Department of Applied Economics, School of Economics, University of Cape Coast, Cape Coast, Ghana; 5 School of Allied Health, Exercise & Sports Sciences, Faculty of Science & Health, Charles Sturt University, Albury, New South Wales, Australia

**Keywords:** Specialist health services, Older adults, Rural health services, Chronic conditions

## Abstract

**Background:**

Limited access to specialist medical services is a major barrier to healthcare in rural areas. We compared rural-urban specialist doctor consultations outside hospital by older adults (≥ 60 years) across South Australia.

**Methods:**

Cross-sectional data were available from the South Australia’s Department of Health. The Modified Monash Model (MM1-7) of remoteness was used to categorize data into rural (MM 3–4), remote (MM5-7), and urban (MM1-MM2) of participants in urban and non-urban South Australia. The analysis was conducted on older adults (*n* = 20,522), self-reporting chronic physical and common mental health conditions.

**Results:**

Specialist doctor consultation in the past 4 weeks was 14.6% in our sample. In multivariable analysis, increasing age (odds ratio 1.3, 95% CI: 1.2-1.4), higher education (odds ratio 1.5, 95% CI: 1.3-1.9), physical health conditions [diabetes (odds ratio 1.2, 95% CI: 1.1-1.3); cancer (odds ratio1.8, 95% CI: 1.7-2.0); heart disease (odds ratio 1.9, 95% CI: 1.6-2.1)], and common mental disorders [depression (odds ratio 1.3, 95% CI: 1.1-1.5); anxiety (odds ratio 1.4, 95% CI: 1.1-1.6)] were associated with higher specialist care use. Specialist care use among rural (odds ratio 0.8, 95% CI: 0.6–0.9), and remote (odds ratio 0.8, 95% CI: 0.7–0.9) older people was significantly lower than their urban counterparts after controlling for age, education, and chronic disease.

**Conclusion:**

Our findings demonstrate a disparity in the use of out of hospital specialist medical services between urban and non-urban areas.

## Abstract (significance)

**What is already known**

• Specialist care is crucial in chronic disease management

• Ageing is associated with higher care needs, including specialty care

• There is limited evidence relating to factors associated with specialist services use in old age

**What this paper adds**

• This study demonstrates a disparity in the use of specialty services between urban and rural environments

• Beside availability of specialty services, socioeconomic factors are associated with specialist care visits

**Application of study findings**:

• Interprofessional care coordination could be a vital resource in chronic health management

• Appropriate specialty care provision and use in rural areas could help reduce the rural-urban health disparities.

## Background

Specialist doctors provide diagnostic and treatment services for specific ailments and help in managing chronic health conditions. Specialist medical services include geriatric medicine, rheumatology, neurology, urology, and among others. Older people (60 + years) constitute 16.5% of the Australian population and are the greatest users of specialist health services [1]. In 2019–2020, about 14.6 million older adults were treated by specialists in the country, which constituted almost 1 in 2 (46%) specialist claims (Medicare-subsidised consultations) [2]. Specialist care services (particularly geriatric services) are crucial for chronic health management to ensure improved quality of life and independent living in old age [3]. However, specialist care services access for older people in rural and remote communities is a concern in many countries [4].

There is expanding research on the impact of geographical location on health services use [5, 6], and health outcomes [7]. Where people live impact their health status, health behaviour, and healthcare access [8]. Rural older adults have reduced access to needed health services, increasing their risk of experiencing poorer health outcomes [9, 10]. For example, limited access to geriatric support and mental health services has been described in Australian rural care system [9, 11]. Additionally, several social determinants of health including education, income, social support, and housing may determine older people access and use of health services in rural locations [8, 12].

Rural people experience poorer health outcomes compared to their urban counterparts, largely due to disadvantages in social determinants of health [13]. Social determinants of health are the factors that impact individuals’ health and wellbeing, which includes locations where people were born, live, work and age, as well as the accessibility and responsiveness of health services to their needs [14–16]. Rurally living is also linked with lower levels of socioeconomic status such as lower education, lower household incomes and both are associated with higher risk of multiple chronic conditions and poor health outcomes [17]. One would hypothesize that, with higher rates of chronic diseases, patients living in rural and remote areas would require higher specialist medical services.

In Australia, rural-urban difference in health services access and outcomes such as General practice (GP) visits, hospitalizations, and Emergency department (ED) visits have been previously reported. While the use of GP services is largely similar across regional and urban areas [9, 18], rural and remote Australians have higher rates of hospitalizations, poorer access to, and use of, primary health services [19] Even though, these reports have highlighted the access gaps and health disparities between rural and urban locations, there is limited understanding of rural-urban differences in specialist doctor visits among older adults, including determining factors of specialist care services use.

Over the past decades, continued research effort has been devoted in developing and refining models of health services access and use to inform policy initiatives. A widely used framework is the Andersen [20] model, which has been applied to understand health service use among vulnerable populations such as older adults. This model suggests that personal health services use balances on three functional domains; predisposing factors—non-modifiable biological factors (e.g., age, sex), enabling factors—facilitators and resources (e.g., location, social support, income, health literacy, belief systems) and need variables—subjective and objective health status (e.g., health status, perception of illness) [20]. The utility of the model in understanding specialist medical service use has not been previously studied.

Based on the Anderson model of health services use, this study proposes that older adults’ use of specialist doctor services is explained by sex and age (predisposition); educational attainment -as proxy to income/wealth, and location (enabling factor); and diagnosed health conditions (need variable). Our study aims to compare self-reported specialist doctor visits outside hospital by older adults (≥ 60 years) across rural and urban South Australia and to validate the factors of specialist visits with the constructs of Andersen’s model in the study sample.

## Materials and methods

### Survey design and research sample

This was a secondary analysis of the interview and/or data from the South Australia’s Population Health Survey—a statewide population health survey designed in the year 2003. This population health survey has undergone a slight modification (addition of variables/questions) and a name changed from South Australian Monitoring and Surveillance System (SAMSS) to South Australian Population Health Survey (SAPHS) in the year 2018. The data was obtained from the South Australian Department of Health. This cross-sectional survey (SAPHS) draws a sample of approximately 7000 from listed households in the Electronic White Pages (EWP) across the state every year [21]. Through random digit dialling, the survey uses a dual overlapping sampling strategy (70% mobile, 30% landline) [21]. This sampling technique is intended to include a representative sample of the population. The only inclusion criterion is being a resident of South Australia with access to a telephone and the average response rate of this survey is 69% [21]. The survey monitors disease burden, access issues, and other critical health concerns of South Australians at regular intervals [9, 22]. Hence, the data provide pertinent retrospective population health information to help in designing policy interventions that are more responsive and focused to meet the health needs of South Australians [22].

Each interview lasts for about 15–20 min. Interviews are conducted in English by trained interviewers and responses are recorded using computer-assisted interviewing (CATI) technology. This system is highly effective in collecting high-quality data on rural and remote communities where the costly conduct of face-to-face surveys has led to underrepresentation of rural cohorts in prior studies [18]. Further information on the survey’s objectives, methodologies, and initial findings can be found elsewhere [22]. Also, the questionnaire for this survey can be found following the link https://www.preventivehealth.sa.gov.au/evidence-data/about-our-data-collections/sa-population-health-survey.

#### Geographical classification

The Modified Monash Model (MMM) was used for rural-urban and remote categorisations. The Australian Department of Health developed the modified Monash model geographical classification system using data on population and remoteness from the Australian Bureau of Statistics (ABS) [23]. Based on ABS data, the model assigns a remoteness index (MM1-MM7) to various postcodes across Australia [24]. We designated postcodes as urban (MM1-MM2), rural (MM3-MM4) and remote (MM5- MM7) based on their population densities and proximity to major cities and services. It is important to emphasize that MM5 is originally classified as small rural town. However, due to low response frequencies for MM6 and MM7, MM5-MM7 were grouped together as small rural-remote to enhance statistical power for the analysis.

#### Measures

##### Sociodemographic variables

Baseline demographic characteristics such as age, educational attainment, and gender were included in the analyses. Age in years was categorized into three groups including 60–69, 70–79, and 80 or above. Education was assessed in three categories namely basic/primary, high school, and diploma or above (Table 1).

**Table 1:** Participants characteristics

Sample Characteristic	Categories	SPC *N* (%)	Chi-square (*p*-value)
Gender	Male	1254(15.5)	8.537(0.003)
	Female	1750(14.1)	
Education	Basic	198 (12.4)	45.971(0.001)
	High School	1383(13.4)	
	TAFE/Diploma/Degree	1416(16.6)	
Age (years)	60–69	1156(13.0)	33.709(0.001)
	70–79	1140(15.8)	
	80 or above	708 (16.1)	
Location	Urban	2218(15.7)	44.082(0.001)
	Rural	294 (12.3)	
	Remote	492 (12.2)	

SPC = specialist care use; TAFE=  Technical and further education

##### Specialist health services use

Specialist doctor visit was assessed with the item “In the last four weeks, have you used Specialist doctor services (not in hospital)”, with binary response categories 0 = no and 1 = yes. It is important to note that this question was not intended to include outpatient specialist services that took place in hospitals.

##### Physical health conditions

Survey participants self-reported common doctor diagnosed health conditions. The reported medical conditions analysed in this study includes diabetes, heart attack, heart disease, and cancer.

##### Mental health conditions

Like physical health conditions, participants indicated the presence of any doctor diagnosed mental health condition and/or ongoing treatment for a mental health condition.

### Ethics statement

The survey adopts a simple procedure to inform potential participants about the survey objectives and participation processes. A standardized introduction is read out to participants regarding who is calling, the purpose of the survey, confidentiality and that the survey is voluntary. Participants are asked if they reside in South Australia and for their postcode. Once a person has consented to participate, the individual may choose to be contacted at an alternative time or day that is most suitable to them. Ethics approval was granted by South Australia Department of Health and Wellbeing’s Human Research Ethics Committee received ethics approval from the relevant institutional review committee (HREC/18/SAH/89) for the analysis of SAPHS data in this study.

### Statistical analysis

All analyses were performed using IBM SPSS software version 24.0 (IBM Corp., Armonk, New York, NY, USA) and statistical significance was set at *p* < 0. 05. Frequencies and percentages were used to describe demographic characteristics of the survey participants. Pearson Chi-Squared statistic assessed rural-urban differences in the use of specialist doctor services. Differences in the use of specialist services by demographic variables and health conditions (self-reported diagnosed health conditions ) were similarly determined by Chi-Squared test. Multivariable logistic regression models were used to explore the effects of explanatory variables on specialist services use, mutually adjusting for other variables in the model.

## Results

Table 1 shows characteristics of the participants. Of the 20,522 (urban = 13,498; rural = 2981; remote = 4043) participants analyzed in this study, 60.7% were female and the mean age was 72.33 years (SD = 8.34), with the age range 60–69 constituted the majority (42.9%). Older adults in urban Adelaide reported higher educational qualification (45.6% with diploma or degree certificate) than rural and remote participants.

### Patterns of specialist doctor visits outside hospital

Prior to the survey, 14.6% of the participants had at least ever-consulted a specialist doctor within the past 4 weeks. Participants aged 80 years or over (16.1%) accessed more specialist doctor services compared to those in the 60–69 (13%) year age group (X^2^ = 33.709, *p* < .001. (Table 1). Older adults who had completed a diploma or degree course (16.6%) accessed more specialist services than the proportions with high school (13.4%) and basic school (12.4%) qualifications (X^2^ = 45.971, *p* < .001). There was a statistically significant difference in specialist health services use by geographical location. Greater proportion of urban older people (15.7%), than rural, and remote (Both 12. %) had visited a specialist doctor prior to the survey (X^2^ = 44.082, *p* < .001).

Specialist doctor visits in the past 4 weeks were more common with the presence of diagnosed physical conditions (diabetes, cancer, heart disease) and common mental health conditions (depression and anxiety) across urban, rural, and remote locations. However, the proportion reporting specialist visits among rural and remote older people with physical health conditions and common mental health disorders was lower than that observed in urban locations. For instance, there was a statistically significant difference in specialist consultations for those with heart disease in urban = 25.6%, rural = 21.2%, and remote = 21.5% locations (*p* < .001). The geographical distribution of specialist doctor visits with diabetes were urban = 15.2%, rural = 11.9%, and remote = 11.7%. Similar significant differences between urban and remote locations were observed for cancer (23.3% vs. 17%), anxiety (21.1% vs. 15.7%), and depression (20.4% vs. 17.7%) (Table 2).

**Table 2:** Health conditions and specialist service use across South Australia

Conditions	Categories	SPC *N* (%)	Chi-square (*p*-value)	Urban	Rural	Remote	*p*-value
Diabetes	No Yes	2432(14.2) 568(17.1)	18.895(0.001)	1809(15.2) 406 (18.5)	232(11.9) 62 (14.3)	391(11.7) 100(14.4)	(*p* < .001)
Heart Disease	Yes No	460 (24.4) 2544(13.6)	158.735(0.001)	346(25.6) 1872(14.7)	43 (21.2) 251(11.5)	71 (21.5) 421(11.3)	(*p* < .001)
Cancer	No Yes	1952(12.5) 1046(21.3)	231.193(0.001)	1426(13.3) 787(23.3)	195(10.8) 98 (16.6)	331(10.7) 161(17.0)	(*p* < .001)
Anxiety	Yes No	200(20.1) 2804(14.4)	24.972(0.001)	148(21.1) 2070(15.5)	25(20.3) 269(11.9)	27(15.7) 465(12.00	(*p* < .001)
Depression	Yes No	237 (19.8) 2767(14.3)	26.804(0.001)	171 (20.4) 2047(15.4)	29 (19.0) 265(11.8)	37 (17.7) 455(11.9)	(*p* < .001)
Mental health treatment	Yes No	461(19.2) 2537(14.0)	44.786(0.001)	334(20.1) 1880(15.2)	46 (15.6) 246(11.8)	81 (18.0) 411(11.5)	(*p* < .001)

SPC = specialist care use

Table 3 presents the results from the multivariable regression model. Increasing age, higher educational attainment, physical health conditions, and common mental disorders were independently associated with a greater likelihood of using specialist care services. Compared to the urban participants, the rural 0.8, 95% CI: 0.6–0.9, *p* = .001), and remote; 0.8, 95% CI: 0.7–0.9, *p* = .001) older adults were less likely to use specialist doctor services, after adjusting for age, gender, education, and any present health condition.

**Table 3:** Association between demographic variables, chronic conditions, and specialist doctor visits

	SPC Use	*p*-value
**Gender**		
Male	1.0	0.331
Female	0.9 (0.8-1.0)	
**Age (years)**		
60–69	1.0	0.001
70–79	1.3 (1.1–1.4)	
80 or above	1.3 (1.2–1.4)	
**Education**		
Basic/Primary school	1.0	0.001
High School	1.2 (1.0-1.4)	
TAFE/Diploma/Degree	1.5 (1.3–1.9)	
**Diabetes**		
No	1.0	0.001
Yes	1.2 (1.1–1.3)	
**Cancer**		
No	1.0	0.001
Yes	1.8 (1.7-2.0)	
**Heart disease**		
No	1.0	0.001
Yes	1.9 (1.6–2.1)	
**Depression**		
No	1.0	0.001
Yes	1.3 (1.1–1.5)	
**Anxiety**		
No	1.0	0.001
Yes	1.4 (1.1–1.6)	
**Rurality**		
Urban	1.0	0.001
Rural	0.8 (0.7–0.9)	
Remote	0.8 (0.7–0.9)	

SPC = specialist care use;  TAFE= Technical and further education

## Discussion

Our study investigated rural-urban differences in specialist doctor consultations outside hospital settings for rural older adults and to understand the associated factors based on Andersen’s behavioural model of health service use. In this study, the use of specialist care services outside hospital among older adults was 14.6%. Increasing age, higher educational attainment, diagnosed physical health conditions and common mental disorders were independently associated with higher specialist care use. There was significant regional disparity in specialist health services use with older adults in rural locations using less specialist services out of hospital compared to older adults in urban locations.

Specialist medical services are critical to chronic disease management, especially in older adults with complex healthcare needs. Rurality was found to be independently associated with less likelihood of specialist care visits. The rural-urban disparity in specialist doctor visits found in this study buttresses the fact that older people in rural Australia experience poorer access to, and use of, needed health services [25, 26]. This result corroborates a similar international study where specialist care utilization was higher in the Finnish capital- Helsinki than regional Finland [12].

Chronic health conditions including diabetes, cancer, heart disease and mental illness were associated with higher odds of non-hospital specialist doctor visits in our study. Schulz, Czwikla [27] have highlighted that disease burden tends to explain differences in the use of medical specialist services among older adults in Germany. Of note, older people with anxiety for instance, were more likely to visit specialists if they were in an urban area (25%) as opposed to a remote community (21%). This may reflect the longstanding challenges in accessing specialised services in Australian rural environments [11, 28]. Greater supply of health services facilitates timely and appropriate services use [29]. This result could also mean two things: First, the underutilization may be contributing to the higher burden of multimorbidity earlier reported elsewhere [9, 30]. Second, the lower utilization is a good thing as it may reflect a broader scope of cost-effective practice by rural generalists saving patients from expensive visits to a specialist. It is, however, worth noting that the balance of probabilities is towards the first option.

Consistent with gerontological studies of health services use (e.g. [31–33], we found that increasing age and higher educational attainment demonstrated associations with specialist doctor visits. Specifically, older people who were 70–79 years and 80 years or above were more likely to visit specialist doctors outside the hospital. It is likely that the more frequent visits to specialist doctor among those 70 years and above is due to their higher burden of diseases [34]. Our findings further reinforce the associations between increasing age, multimorbidity, and high healthcare demand. Higher education may enable individuals to afford private specialist services due to its associations with higher incomes [12]. According to the ‘Rural and Remote Health’ report [25, 35], social determinants such as income and education partly account for health inequalities such as access to specialists in rural and remote areas.

Our results validate the theoretical constructs of the Andersen’s behavioural health model. Age and gender (predisposing factors) demonstrated associations with specialist services use. Many international studies [36, 37] analyzing health services use through the behavioral model have made similar conclusions. Similarly, having chronic health conditions (need factors) independently predicted specialists visits. Chronic health conditions have been verified in similar studies as need variable associated with health services utilization among older adults [36, 38, 39]. Lastly, rurality and education; specified under the enabling construct of Andersen’s model showed associations with specialist services use. As discussed earlier, education may improve socioeconomic status of an individual and as well broaden their knowledge base to make health services more accessible to them. Conversely, rurality may serve as a barrier to health services due to lower levels of education, limited healthcare resources and the concept of distance decay [40].

After several years of research and policy efforts, it is worrying to note that rural and remote populations continue to grapple with unmet health services need [19, 25]. Gruen and colleagues [28] argue that most of the illnesses responsible for rural population’s higher morbidity and mortality would ordinarily be managed with greater accessibility to specialist services.

### Limitations

This study is one of the first to investigate rural-urban differences in specialist care services use and validate the factors associated with specialist care visits with the Andersen’s behavioural model of health service use. The results of this study should be interpreted along some limitations. First, this study used a cross-sectional data, and causality cannot be determined. Second, older people accessing specialist services through hospitals is not assessed in this study because we did not have access to that data. Hence, we do not know the overall use of specialist services (e.g., hospital outpatient services). Third, even though, there is information about diagnosed conditions, we do not know anything about the severity of these conditions which have an important impact on the need for health services use. Again, the question used in the survey relative to specialist visit is quite ambiguous. Lastly, the quantitative nature of the study limited us to capture the normative views of older adults in relation to factors influencing specialist doctor visit. As a result of this, a mixed methods study on non-hospital specialist doctor consultation is warranted.

## Conclusion

We have demonstrated that rural, and remote older adults in South Australian use specialist services fewer than their urban counterparts. This may have contributed to the higher burden of diseases among older people in rural locales. Conversely, the lower use of specialist medical services may indicate cost-effective practice by rural generalists saving older patients expensive visits to specialists. It is worthy to note that the balance of probabilities is towards the limited access to specialist services and lower socioeconomic levels in rural and remote Australia. The findings of this study warrant further exploration to improve access to evidence-based specialist care interventions throughout the healthcare system, but especially in rural settings. A potential approach could be interprofessional care coordination that strengthens rotation of specialists to rural environments, providing on-site consultations and training local practitioners.

## Data Availability

The datasets generated and/or analysed during the current study are available at [South Australia Department of Health] Repository. https://data.sa.gov.au/data/dataset/c1a77b0e-84df-4ac7-9f4d-bf1f6b92d265.
